# Viral RNA Genomes Identified from Marine Macroalgae and a Diatom

**DOI:** 10.1264/jsme2.ME20016

**Published:** 2020-06-17

**Authors:** Yuto Chiba, Yuji Tomaru, Hiromori Shimabukuro, Kei Kimura, Miho Hirai, Yoshihiro Takaki, Daisuke Hagiwara, Takuro Nunoura, Syun-ichi Urayama

**Affiliations:** 1 Laboratory of Fungal Interaction and Molecular Biology (donated by IFO), Department of Life and Environmental Sciences, University of Tsukuba, 1–1–1 Tennodai, Tsukuba, Ibaraki, 305–8577, Japan; 2 Faculty of Science, International College of Arts and Science, Yokohama City University, 22–2 Seto, Kanazawa-ku, Yokohama 236–0027, Japan; 3 Japan Fisheries Research and Education Agency, National Research Institute of Fisheries and Environment of the Inland Sea, 2–17–5 Maruishi, Hatsukaichi, Hiroshima 739–0452, Japan; 4 Faculty of Agriculture, Saga University, Honjo-machi 1, Saga 840–8502, Japan; 5 Super-cutting-edge Grand and Advanced Research (SUGAR) Program, JAMSTEC, 2–15 Natsushima-cho, Yokosuka, Kanagawa 237–0061, Japan; 6 Microbiology Research Center for Sustainability (MiCS), University of Tsukuba, 1–1–1 Tennodai, Tsukuba, Ibaraki, 305–8577, Japan; 7 Research Center for Bioscience and Nanoscience (CeBN), Japan Agency for Marine-Earth Science and Technology (JAMSTEC), 2–15 Natsushima-cho, Yokosuka, Kanagawa 237–0061, Japan

**Keywords:** RNA virus, algae, dsRNA

## Abstract

Protists provide insights into the diversity and function of RNA viruses in marine systems. Among them, marine macroalgae are good targets for RNA virome analyses because they have a sufficient biomass in nature. However, RNA viruses in macroalgae have not yet been examined in detail, and only partial genome sequences have been reported for the majority of RNA viruses. Therefore, to obtain further insights into the distribution and diversity of RNA viruses associated with marine protists, we herein examined RNA viruses in macroalgae and a diatom. We report the putative complete genome sequences of six novel RNA viruses from two marine macroalgae and one diatom holobiont. Four viruses were not classified into established viral genera or families. Furthermore, a virus classified into *Totiviridae* showed a genome structure that has not yet been reported in this family. These results suggest that a number of distinct RNA viruses are widespread in a broad range of protists.

Marine macroalgae are classified into multiple eukaryotic lineages that are phylogenetically distinct from each other. For example, green algae and red algae belong to Chloroplastida and Rhodophyceae in Archaeplastida, respectively, while brown algae belongs to Stramenopiles in the SAR supergroup (*i.e.*, Stramenopiles, Alveolata, and Rhizaria) (Adl *et al.*, 2012). Although diatoms, which belong to the Stramenopiles lineage, are recognized as microalgae, they sometimes form macrocolonies. These marine macroalgae are considered to be a type of holobiont, a functional ecosystem, because of the relationship between macroalgae and diverse microorganisms. Thus, to elucidate the functional relationship between macroalgae and associated microbes, metagenomic approaches have been applied to macroalgal holobionts (Egan *et al.*, 2013; Lachnit *et al.*, 2015).

Recent studies suggested that viruses, in addition to cellular microorganisms, are involved in the homeostasis and evolution of holobiont systems (Marhaver *et al.*, 2008; Barr *et al.*, 2013; Bruwer *et al.*, 2017; Thurber *et al.*, 2017). However, viruses in macroalgal holobionts have not yet been examined in detail. To date, two DNA viruses, *Ectocarpus siliculosus virus 1* (Lanka *et al.*, 1993) and *Feldmannia species virus* (Henry and Meints, 1992), have been isolated from marine macroalgae. Only two virome analyses have been conducted on RNA viruses (Lachnit *et al.*, 2015; Waldron *et al.*, 2018). One analysis involved the identification of partial viral RNA sequences in the Tombus-Noda, Bunya-Arena, and Narna-Levi clades from a metatranscriptome in a mixture of brown algae (*Fucus serratus*) (Waldron *et al.*, 2018). The other consisted of a combination of virus particle purification and RNA sequencing from two individuals of red algae (*Delisea pulchra*): the sequences of members of *Totiviridae*, *Partitiviridae*, and *Picornavirales* were commonly detected (Lachnit *et al.*, 2015). In addition, unassigned mitochondria-associated dsRNA and chloroplast-associated dsRNA have been reported from the green alga *Bryopsis cinicola* (Koga *et al.*, 1998, 2003).

Complete genome sequences are essential for understanding the diversity and evolution of RNA viruses (Shi *et al.*, 2016; Dolja and Koonin, 2017; Shi *et al.*, 2018; Wolf *et al.*, 2018). However, conventional RNA sequencing methods are technically challenging. Thus, several novel molecular techniques have been developed to obtain complete genome information on RNA viruses (Lambden *et al.*, 1992; Vreede *et al.*, 1998; Maan *et al.*, 2007; Potgieter *et al.*, 2009; Darissa *et al.*, 2010; Inoue *et al.*, 2010). Fragmented and primer ligated dsRNA sequencing (FLDS) is a novel method that provides full-length viral RNA genome segments and reconstructs putative complete genomes (Urayama *et al.*, 2016, 2018, 2020). Using this method, a sequencing library is constructed from cellular dsRNA molecules consisting of dsRNA virus genomes and replicative intermediates of ssRNA viruses (Morris and Dodds, 1979), and the entire sequences of dsRNA molecules are reconstructed *in silico*.

In the present study, to investigate the distribution and diversity of RNA viruses associated with marine macroalgae holobionts, FLDS was applied to three marine macroalgae holobionts that contained sufficient amounts of dsRNA to be observed by agarose gel electrophoresis. We successfully obtained the putative complete genome sequences of six novel RNA viruses and identified a highly novel RNA virus with a new genome structure that was not recognized based on partial sequence information.

## Materials and Methods

### Sample collection

Based on morphology, we collected marine macroalgae in front of the National Research Institute of Fisheries and Environment of Inland Sea, Japan Fisheries Research and Education Agency (34°16'29.2"N 132°15'57.7"E) on February 18, 2016. After carefully washing the samples with autoclaved seawater (121°C, 15‍ ‍min) that was passed through polycarbonate membrane filters with a pore size of 0.2 μm (GE Healthcare Life Sciences), excess water was removed using paper towels. Treated samples were stored at –80°C until analyzed.

### RNA extraction

Macroalgae samples 1–6 (0.40, 0.46, 0.37, 0.30, 0.81, and 0.96‍ ‍g wet weight, respectively) were disrupted in liquid nitrogen with a mortar. Regarding dsRNA purification, samples were suspended in 2× STE (0.2 M Tris–HCl, 0.2 M NaCl, and 2‍ ‍mM EDTA, pH 6.8) containing 0.1% (v/v) β-mercaptoethanol, and total nucleic acids were manually extracted with SDS-phenol. dsRNA was purified twice through a Poly-Prep Empty Chromatography Column (Bio-Rad) and microspin column (empty Bio-spin column; Bio-Rad) containing cellulose powder (Cellulose D; ADVANTEC). To remove the remaining DNA and ssRNA, eluted dsRNA was further treated with amplification grade DNase I (Invitrogen) and S1 nuclease (Invitrogen) as described previously (Urayama *et al.*, 2018).

To purify ssRNA, part of the pulverized sample was treated with a TRIzol Plus RNA Purification Kit (Invitrogen) according to the manufacturer’s protocol. The total ssRNA fraction was treated with amplification grade DNase I (Invitrogen) and purified using RNA Clean & Concentrator-5 (Zymo Research).

### Library construction and sequencing

cDNA libraries were constructed from purified dsRNA and ssRNA as described previously (Urayama *et al.*, 2018). In brief, dsRNA obtained from each sample was converted into a cDNA library using the FLDS method. The U2 primer was ligated to the 3′ end of fragmented dsRNA, and cDNA was synthesized using the SMARTer RACE 5′/3′ Kit (Takara Bio) with the U2-comp primer. Regarding total RNA-seq, ssRNA was converted into a cDNA library using the SMARTer Universal Low Input RNA Kit according to the manufacturer’s protocol (Takara Bio). After PCR amplification, cDNA was fragmented by an ultrasonicator (Covaris S220). Illumina sequencing libraries were then constructed using KAPA Hyper Prep Kit Illumina platforms (Kapa Biosystems) and evaluated using the KAPA Library Quantification Kit (Kapa Biosystems). The libraries were sequenced using the Illumina MiSeq v3 Reagent Kit (600 cycles) with 300-bp paired-end reads on the Illumina MiSeq platform.

### Data processing

The raw sequence reads obtained from FLDS libraries were processed to remove low-quality, adaptor, rRNA, and low-complexity sequences as described previously (Urayama *et al.*, 2018). The remaining reads were subjected to *de novo* assembly using CLC GENOMICS WORKBENCH version 11.0 (CLC Bio) with the following parameters: a minimum contig length of 500, word value set to auto, and bubble size set to auto. To obtain putative complete viral RNA genomes from FLDS data, the ends of the contigs with at least 250× average coverage were extended using CLC GENOMICS WORKBENCH version 11.0, Genetyx version 14 (Genetyx), and Tablet viewer version 1.19.09.03 (Milne *et al.*, 2010). In our previous study, contigs, for which both ends were termini, were identified as full-length genome segments and viral genomes were reconstructed (Urayama *et al.*, 2018). To analyze the total RNA virome of three marine macroalgae, total FLDS data were used for *de novo* assembly, and the assembled contigs with at least 3× average coverage and 500 nt in length were clustered at 90% identity using VSEARCH (Rognes *et al.*, 2016). The cluster centroid sequences were selected as representative sequences.

The raw sequence reads obtained from total RNA-seq libraries were trimmed as described previously (Urayama *et al.*, 2016). Small subunit (SSU) rRNA sequences were reconstructed from total RNA-seq reads with EMIRGE, which is an iterative template-guided assembler that relies on a database of 16S rRNA genes (Miller *et al.*, 2011). As the reference database, the SILVA SSU version 132 database was downloaded by running emirge_makedb.py (option: -i 0.99), and we added the mitochondrial 16S rRNA gene sequences of morphologically identified macroalgae species (*Scytosiphon lomentaria* and *Ectocarpus siliculosus*) to the SILVA database because these sequences were absent in the original SILVA database. In the surveillance of RNA-dependent RNA polymerase (RdRp)-coding sequences from total RNA-seq data, trimmed reads were assembled as described above.

RNA viral genes were identified using the BlastX program against the NCBI non-redundant (nr) database with an e-value ≤1×10^–5^. To identify the taxonomic status, the taxonomic information of the top hit virus sequences was used.

### Phylogenetic analysis

The phylogenetic positions of the RNA viruses identified were analyzed based on the deduced amino acid sequences of RdRp genes using the maximum-likelihood method, specifically RAxML (Stamatakis, 2014). Related RdRp sequences were collected and aligned with identified RdRp sequences using MUSCLE (Edgar, 2004) in MEGA6 (Tamura *et al.*, 2013). Ambiguous positions in the alignment were removed using trimAl with the option gt=1 (Capella-Gutiérrez *et al.*, 2009). The best-fitting model of amino acid substitutions was tested in Aminosan (Tanabe, 2011) and judged by the corrected Akaike information criterion (Sugiura, 1978). Bootstrap tests were conducted with 1,000 samplings. FigTree (Rambaut, 2014) was used to illustrate the resulting phylogenies.

### Data accessibility

Datasets supporting the results of the present study are available in the GenBank database repository (Accession Nos. DDBJ: LC521321–LC521329) and Short Read Archive database (Accession No. DDBJ: DRA009245).

## Results

### Major RNA viruses and cellular rRNAs

**Fig. 1.**
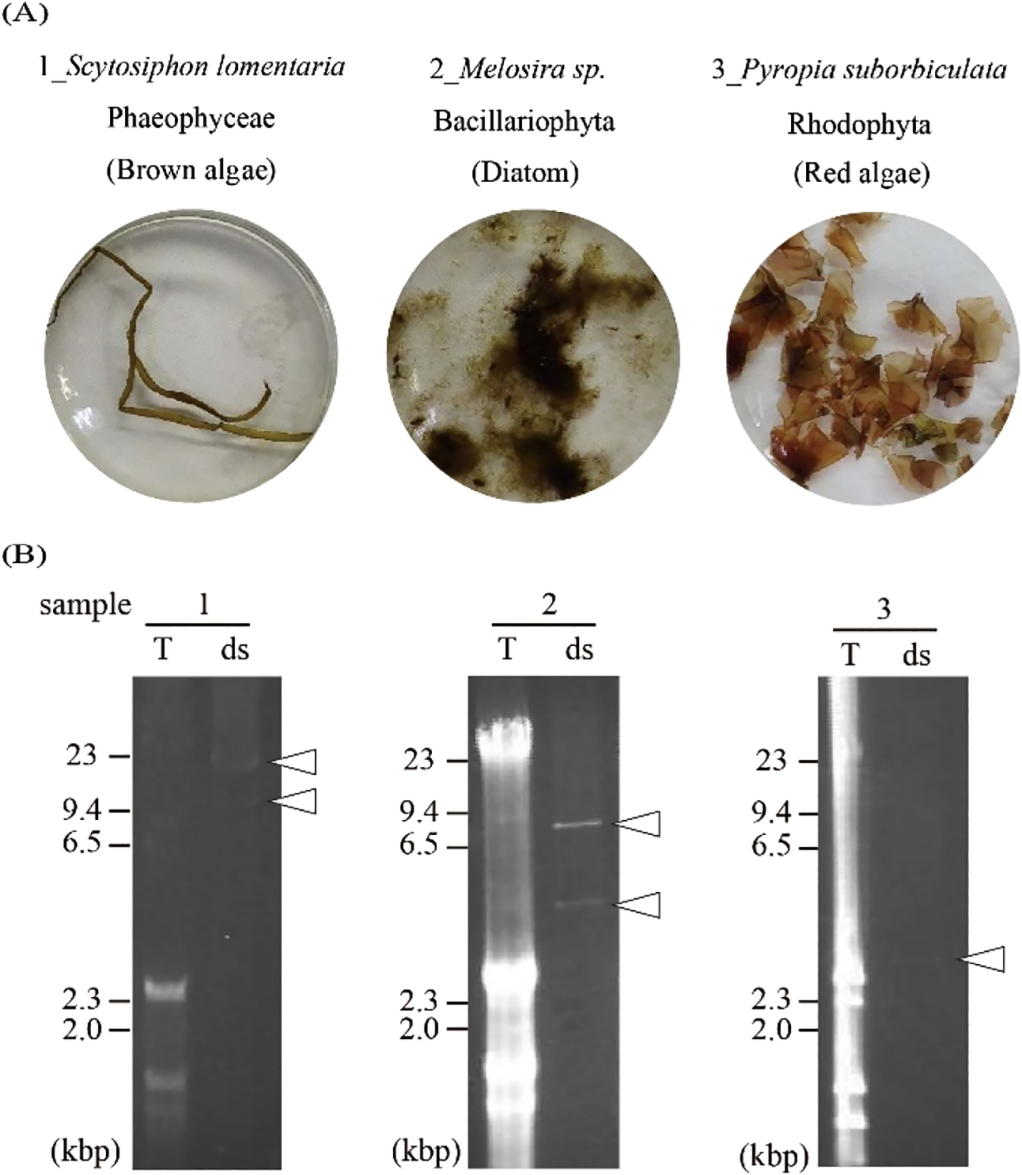
dsRNA-positive marine macroalgae used in the present study. (A) They were identified by morphology and SSU rRNA sequences reconstructed from total RNA-seq data. (B) Agarose gel electrophoresis of the total nucleic acids (T) and dsRNA (ds) of marine macroalgae samples 1–3. Arrowheads indicate dsRNA bands. Nucleic acids were stained with ethidium bromide.

In the surveillance of RNA viruses associated with the marine macroalgae samples, one or two dsRNA band(s) that suggested the presence of RNA viruses were detected (Fig. 1 and Table 1). We then performed FLDS on these dsRNAs and reconstructed viral RNA genomes *in silico* (see Materials and Methods). We found twelve viral segments and eight viral RNA genomes were reconstructed (Table 1 and S1). Among them, two RNA viruses occupied approximately 0.4 and 7% of the reads in each FLDS library. The other six RNA viruses dominated approximately 19–74% of reads in each FLDS library (Table S2). Since the percentage of reads mapped to the genomes of these six RNA viruses was particularly high in each library, we did not expect the presence of other segments of their genomes. Therefore, we conducted a more detailed analysis of the six dominant RNA viruses individually and summarized the entire RNA viromes of these three marine macroalgae, including the minor populations.

To clarify the active holobiont population of samples, SSU rRNA sequences were reconstructed from the reads obtained from total RNA-seq. Their compositions were estimated based on mapped read numbers on the SSU rRNA sequences (Miller *et al.*, 2011). Brown algae, diatoms, and red algae occupied more than 80% of all SSU rRNA reads in the total RNA-seq libraries from samples 1, 2, and 3, respectively (Fig. S1). This result indicated that sample 2 was a diatom holobiont, but not a macroalgae holobiont. Therefore, we designated samples 1, 2, and 3 as a brown algae holobiont, diatom holobiont, and red algae holobiont, respectively (Fig. S1). For example, the SSU rRNA reads of the red algae holobiont included *Pyropia suborbiculata* (red algae, 69.6%), *Pyropia yezoensis* chloroplast (red algae, 24.0%), *Pyropia tenera* (red algae, 5.2%), *Halochlorococcum dilatatum* (diatom, 1.2%), *P. yezoensis* mitochondria (red algae, 0.1%).

### RNA viruses in the brown algae holobiont

**Fig. 2.**
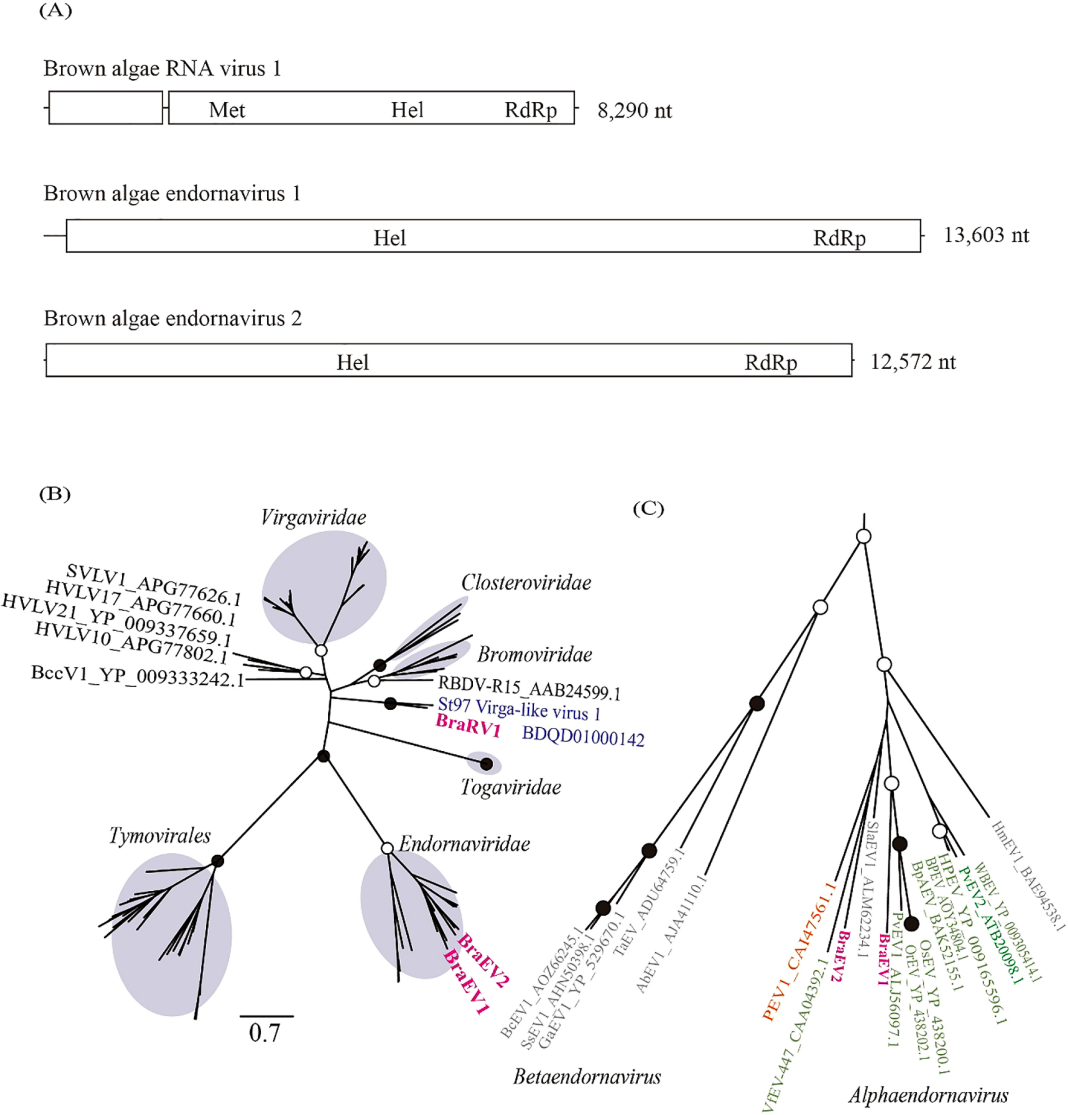
Genome organization and phylogenetic position of RNA viruses identified from a brown algae sample. (A) Predicted ORFs and identified domains: Met, viral methyltransferase superfamily; Hel, viral helicase 1 superfamily; RdRp, RdRP_2 superfamily. (B) Maximum-likelihood tree of RdRp amino acid sequences from representative members of the Hepe-Virga clade (Shi *et al.*, 2016) and three RNA viral sequences obtained in the present study. Open and closed circles represent bootstrap values of 50–90% and ≥90%, respectively. The best-fitting substitution model was [LG+I+G+F]. A virus identified from surface seawater (Urayama *et al.*, 2018) is marked in blue. Brown circles indicate previously established RNA virus families or order. The scale bar represents the number of amino acid substitutions per site. (C) Enlarged view of the phylogenetic tree of *Endornaviridae* in Fig. 2B. Viruses identified from plants are marked in green. Orange and gray indicate oomycete endornavirus and fungal endornavirus, respectively.

Three putative complete genomes (13,603 nt, 12,572 nt, and 8,290 nt) were obtained in the brown algae holobiont (Fig. 2A). Based on a homology search with the predicted amino acid sequence, particularly the open reading frame (ORF) encoding RdRp, the sequences with 13,603 nt and 12,572 nt were named brown algae endornavirus 1 (BraEV1) and brown algae endornavirus 2 (BraEV2), respectively. The 8,290-nt sequence was named brown algae RNA virus 1 (BraRV1). BraEV1 and BraEV2 corresponded to a band of approximately 13‍ ‍kbp, and BraRV1 to a 9-kbp band (Fig. 1, sample 1). A phylogenetic analysis with the RdRp amino acid sequence revealed that BraEV1 and BraEV2 belonged to the established family *Endornaviridae*, and BraRV1 formed a single unclassified deep branch with St97 virga-like virus 1 (Accession: BDQD01000142) (Urayama *et al.*, 2018) identified from surface seawater from the north Pacific Ocean (Fig. 2B and C). Based on the results of a BlastP analysis, the closest isolate of BraRV1 was the plant ssRNA virus, raspberry bushy dwarf virus (RBDV). A stretch of cytosine is present at the 3′ end of many viruses in the family *Endornaviridae* (Okada *et al.*, 2018a). In the present study, the stretch of cytosine was found in BraEV1, but not in BraEV2. The polyproteins encoded by BraEV1 and BraEV2 contained a cysteine-rich region with conserved CXCC motifs shared among several viruses in the family *Endornaviridae* (Okada *et al.*, 2011).

### RNA viruses in the red algae holobiont and diatom holobiont

**Fig. 3.**
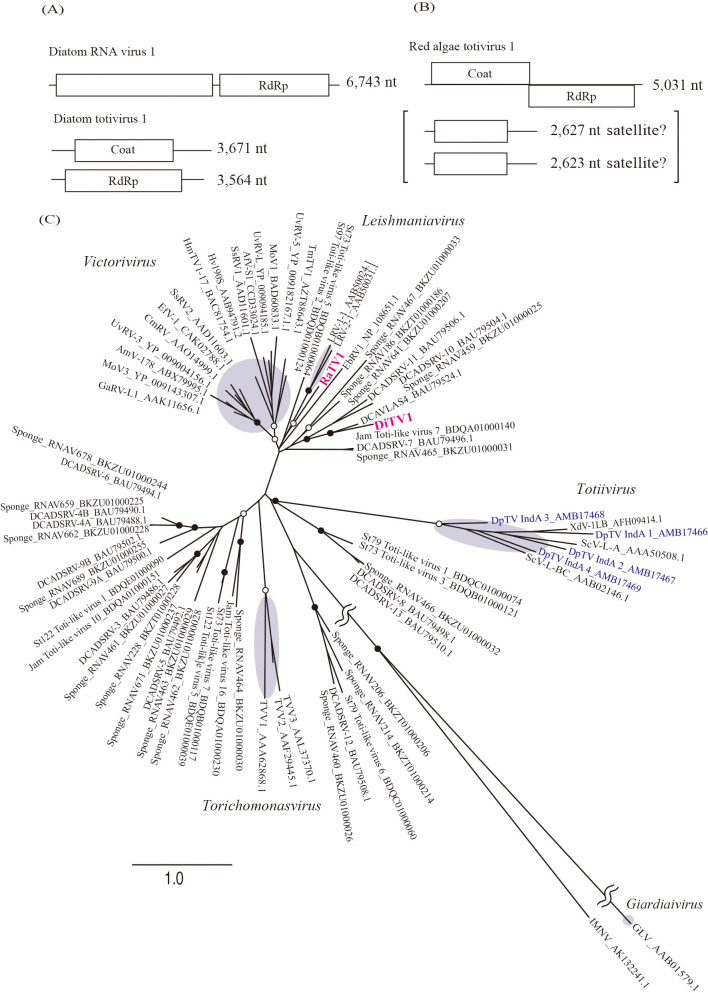
Genome organization and phylogenetic position of RNA viruses identified from diatom and red algae samples. (A, B) Predicted ORFs and identified domains: Coat, Totivirus_coat superfamily; RdRp, RdRP_4 superfamily. (C) Maximum-likelihood tree of RdRp amino acid sequences from representative members of the family *Totiviridae*, two RNA viral sequences obtained in the present study, and their relatives. Open and closed circles represent bootstrap values of 50–90% and ≥90%, respectively. The best-fitting substitution model was [LG+I+G+F]. Viruses identified from red algae (Lachnit *et al.*, 2015) are marked in blue. Brown circles indicate previously established RNA virus genera. The scale bar represents the number of amino acid substitutions per site.

Three viral RNA genomes were obtained from the diatom and red algae holobiont: diatom RNA virus 1 (DiRV1) and diatom totivirus 1 (DiTV1) from the diatom holobiont and red algae totivirus 1 (RaTV1) from the red algae holobiont (Fig. 3A and B). DiRV1 with 6,743 nt appeared to correspond to the 7-kbp band observed in the electrophoresis results (Fig. 1, sample 2). A BlastX search revealed that DiRV1 only had significant similarity with diatom colony associated dsRNA virus 16, an unclassified RNA virus identified from a diatom colony in a tidal pool (Urayama *et al.*, 2016).

However, RaTV1 and DiTV1 were related to non-segmented dsRNA viruses in *Totiviridae* (Fig. 3C and see below). DiTV1 consisted of 3,671-nt and 3,564-nt segments and were named DiTV1 RNA1 and RNA2, respectively. These segments corresponded to the 4-kbp dsRNA band (Fig. 1, sample 2). The terminal sequences of DiTV1 RNA1 and RNA2 were shared with each other (Fig. S2), which is one of the hallmarks of the segmented genome of an RNA virus (Hutchinson *et al.*, 2010). RNA1 and RNA2 were predicted to encode an capsid protein (CP) and RdRp, respectively. In the case of RaTV1, although RNA1 was not detected in gel electrophoresis, RNA2 and RNA3 with 2,627 and 2,623 nt, respectively, appeared to correspond to the 3-kbp band (Fig. 1, sample 3). The terminal sequences were also highly conserved among the three segments (Fig. S2). RaTV1 RNA1 showed the typical ORF structure of a virus in *Totiviridae*. Similar to many viruses in the genus *Victorivirus* (family *Totiviridae*) (Jamal *et al.*, 2019), an overlap region was observed between the CP start and RdRp stop codons (AUGA; nt 2,520 to 2,523). The predicted amino acid sequences of RNA2 and RNA3 did not show significant similarities to known proteins in the NCBI nr database or to conserved motifs in the Pfam database. Since some viruses in *Totiviridae* contain a satellite RNA called M (Tipper and Schmitt, 1991), these RNAs were expected to be satellite RNAs of RaTV1. The results of a phylogenetic analysis showed that DiTV1 and RaTV1 were not classified in any previously established genera in *Totiviridae* (Fig. 3C). To date, viruses classified into *Totiviridae* (DpTV, AMB17466–17469 in Fig. 3C) in *D. pulchra* (red algae) have been reported (Lachnit *et al.*, 2015); however, their phylogenetic position is distinct from DiTV1 and RaTV1.

### Other viral sequences

**Fig. 4.**
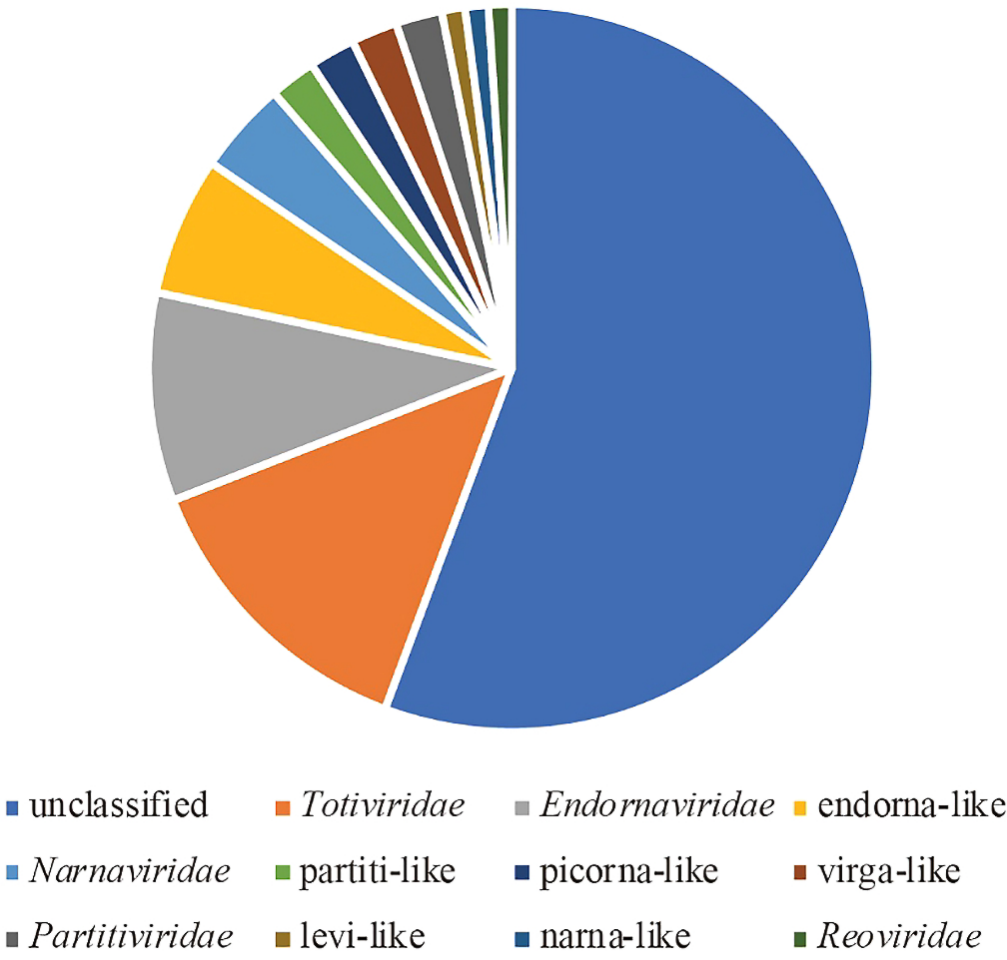
Classification of 98 representative contigs based on taxonomic information on their top hit virus.

**Fig. 5.**
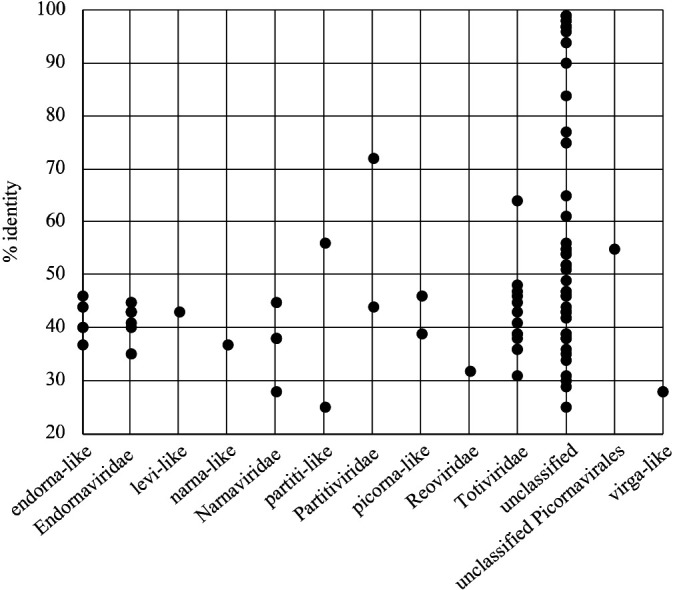
Plot of amino acid % identity for each centroid encoding RdRp to a known viral sequence.

To analyze the total RNA virome, we focused on contigs encoding RdRp. Ninety-eight representative contigs showed significant similarity with RdRp genes in public databases. Based on the Blast top hit sequences, more than 50% of these contigs were designated as unclassified lineages, and the other contigs were identified as members of *Totiviridae*, *Endornaviridae*, endorna-like, or *Narnaviridae* (Fig. 4). In addition, 67% of the RdRp contigs appeared to represent new viral species with less than 50% amino acid identity to the known viral sequence (Fig. 5).

Previous studies reported that the degree of concentrations for viral RNA sequences in dsRNA-seq data differed from that in virus species (Urayama *et al.*, 2016; Decker *et al.*, 2019). We cannot rule out the possibility that some RNA viruses are easier to detect in total RNA-seq than in dsRNA-seq. However, RNA viruses specific for total RNA-seq data were not identified in the present study (data not shown).

## Discussion

In the present study, we identified three dsRNA-positive samples among 6 samples (Fig. S3). The frequency of detectable dsRNA in marine macroalgae holobionts reflected the high infection rate of RNA viruses in fungi and plants and the broad distribution of viral dsRNA in eukaryotes (Koga *et al.*, 1998). The present results suggest that marine macroalgae holobionts harbor diverse RNA viruses and include novel RNA viruses, such as fungi (Kotta-Loizou, 2019), plants (Roossinck *et al.*, 2010; Kamitani *et al.*, 2019), and animals (Shi *et al.*, 2016; 2018; Urayama *et al.*, 2020). Although data are limited, the present results support the hypothesis that protists harbor diverse RNA viruses. To prove this hypothesis, further information is needed on the diversity and distribution of RNA viruses in taxonomically diverse hosts.

Based on phylogenetic analyses of the conserved polymerase, helicase, and methyltransferase motifs (Koonin *et al.*, 1993), RBDV belongs to the ancestral lineage of the family *Bromoviridae* (Isogai *et al.*, 2019). The genome size of BraRV1 is similar to that of viruses in *Bromoviridae* (8.0‍ ‍kb) and RBDV (7.7‍ ‍kb), but differs from those of *Bromoviridae* viruses that have the three-segmented genome. These results suggest that BraRV1 is distantly related to an established RNA virus family and forms a novel RNA virus group with St97 virga-like virus 1. All known viruses in *Totiviridae* harbor undivided dsRNA genomes with two large ORFs (King *et al.*, 2012). The 5′-proximal ORF encodes a CP, and the downstream 3′-proximal ORF encodes an RdRp. Therefore, DiTV1 appears to be a novel member of the family *Totiviridae* with a unique genome structure.

Although the effects of these RNA virus infections are not predictable, some RNA viruses impact the phenotype of host organisms, such as viral toxin production (Magliani *et al.*, 1997) and host toxin production (Okada *et al.*, 2018b), cytological alterations in cellular organelles (Newhouse *et al.*, 1983), and stress tolerance (Marquez *et al.*, 2007). To understand the influence of these widely distributed RNA viruses in marine macroalgae holobionts, we need to isolate and cultivate possible macroalgae hosts and compare virus-infected and virus-free strains. For example, algal strains for culturing laver (nori farming) may be good targets because cultivation systems under laboratory conditions have already been established; however, it currently remains unclear whether these strains harbor RNA viruses. Since difficulties are associated with both the cultivation of host organisms and isolation of viruses from diverse hosts, metagenomics is also required to elucidate the host-RNA virus relationship, as in the case of DNA viruses in diverse marine ecosystems (Silveira *et al.*, 2020). The present results will promote further research on the functions and distribution of RNA viruses in protists, particularly marine macroalgae.

## Citation

Chiba, Y., Tomaru, Y., Shimabukuro, H., Kimura, K., Hirai, M., Takaki, Y., et al. (2020) Viral RNA Genomes Identified from Marine Macroalgae and a Diatom. *Microbes Environ ***35**: ME20016.

https://doi.org/10.1264/jsme2.ME20016

## Supplementary Material

Supplementary Material

## Figures and Tables

**Table 1.** Summary of dsRNA detection and sequence analysis.

Sample name	Category	dsRNA band(s)	Virus name	Family	Length	Average coverage	Accession no.
1	Brown algae	13‍ ‍kbp	Brown algae endornavirus 1	*Endornaviridae*	13,603	18,432.2	LC521321
			Brown algae endornavirus 2	*Endornaviridae*	12,572	8,181.6	LC521322
		9‍ ‍kbp	Brown algae RNA virus 1	unclassified	8,290	15,180.5	LC521323
2	Diatom	7‍ ‍kbp	Diatom RNA virus 1	unclassified	6,743	18,090.4	LC521324
		4‍ ‍kbp	Diatom totivirus 1	*Totiviridae*	3,671	13,489.8	LC521325
					3,564	3,961.4	LC521326
3	Red algae	—	Red algae totivirus 1	*Totiviridae*	5,031	318.8	LC521327
		3‍ ‍kbp			2,627	1,156.3	LC521328
					2,623	531.3	LC521329
