# Supplementary material for: Viral RNA Genomes Identified from Marine Macroalgae and a Diatom
Source: Microbes Environ. 2020 Jun 17;35(3):ME20016. doi: 10.1264/jsme2.ME20016 (PMC7511793; doi:10.1264/jsme2.ME20016)
Supplement: Supplementary file 1 — Supplementary Material [file 35_20016_s1.pdf]

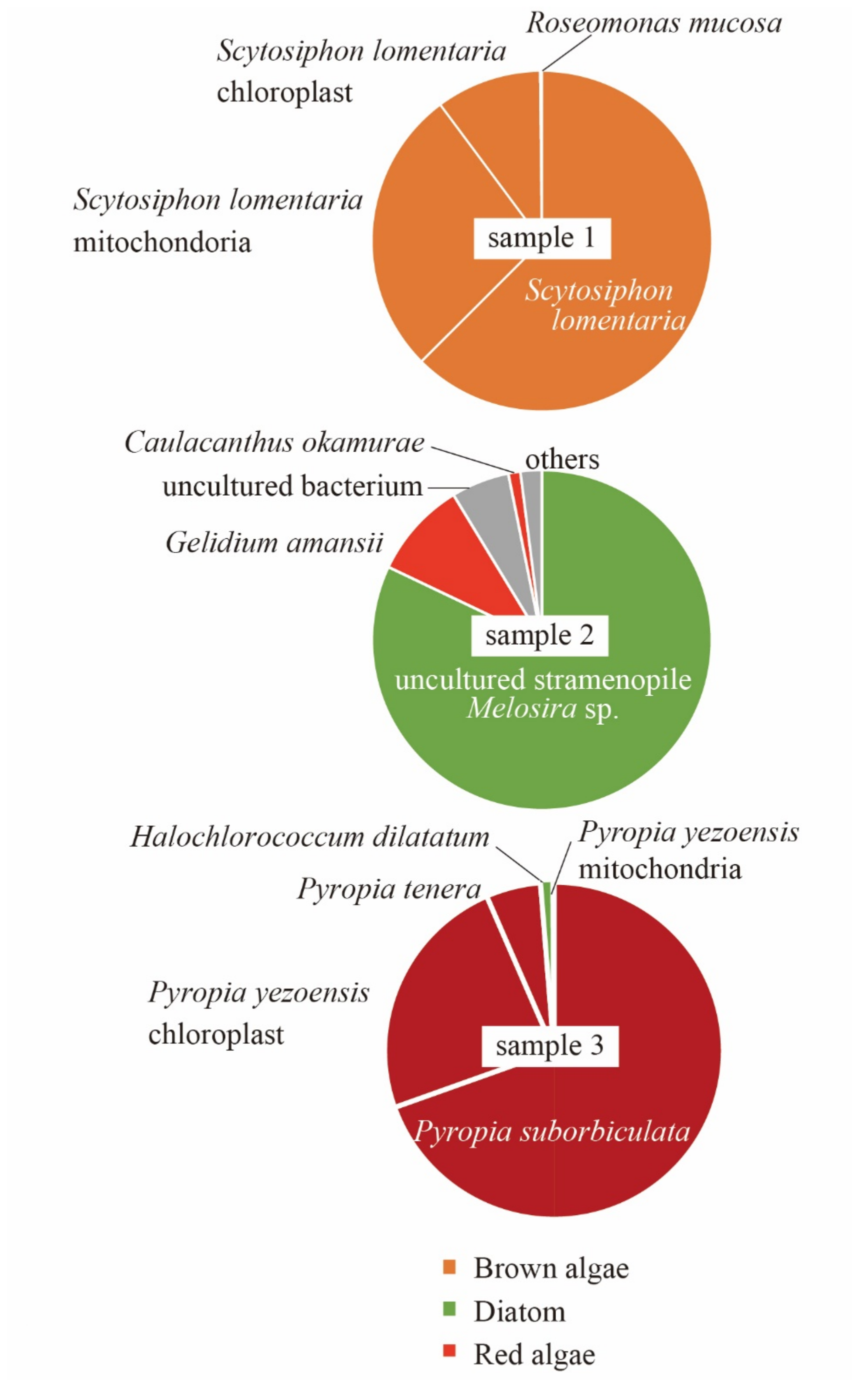

Fig. S1: Relative abundance of sequence reads mapped on the rRNA sequences constructed by EMIRGE. OTUs with relative abundance of 1% or less were summarized as others.

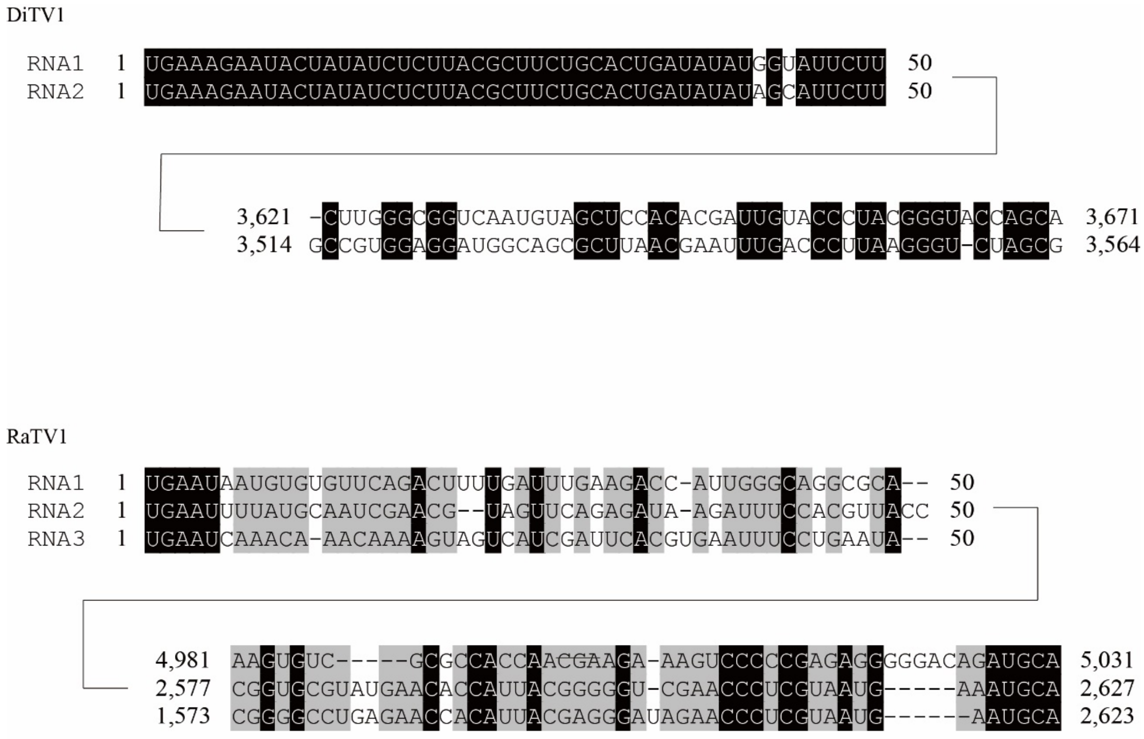

Fig. S2: Multiple alignments of the 5ˈ- and 3ˈ-terminal regions of the coding strands of RNA segments.

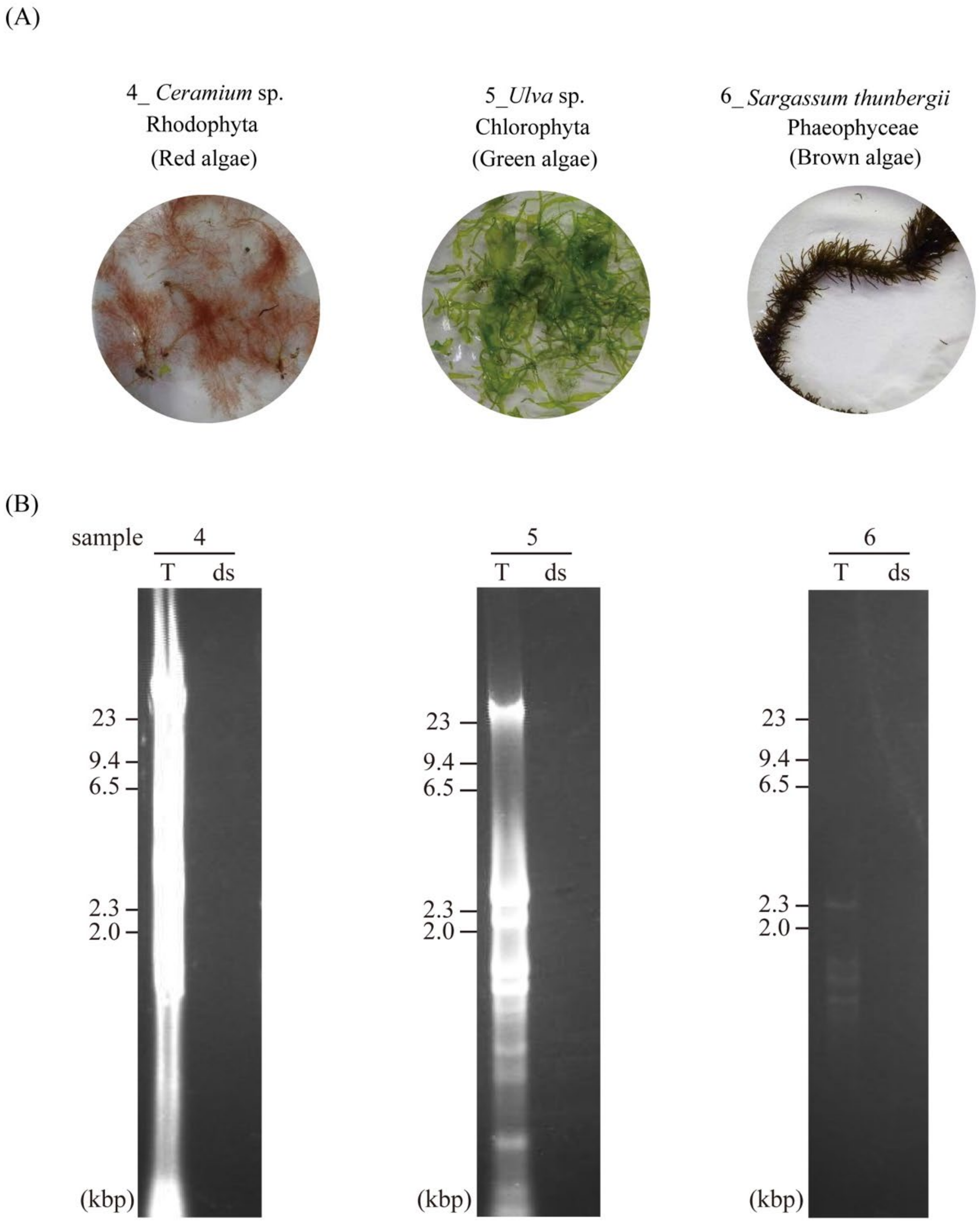

Fig. S3: dsRNA-negative marine macroalgae used in this study. (A). Identification and morphology of marine macroalgae samples 4-6. They were identified by morphology. (B) Agarose gel electrophoresis of the total nucleic acids (T) and dsRNA (ds) of marine macroalgae samples 4-6. Nucleic acids were stained with ethidium bromide.

Table S1

virus	segment	RdRp domain	dsRNA library			
			total read count	average coverage	% in trimmed reads (segment)	% in trimmed reads (virus)
Brown algae endornavirus 1		Yes	1,035,989	18,432.2	48.5	48.5
Brown algae endornavirus 2		Yes	409,077	8,181.6	19.2	19.2
Brown algae RNA virus 1		Yes	528,797	15,180.5	24.8	24.8
Diatom RNA virus 1		Yes	562,244	18,090.4	50.5	50.5
Diatom totivirus 1	1		219,879	13,489.8	19.8	25.4
	2	Yes	61,980	3,961.4	5.6	
Red algae totivirus 1	1	Yes	6,868	318.8	20.3	74.0
	2		12,403	1,156.3	36.7	
	3		5,730	531.3	17.0	

Table S2

virus	segment	BlastN			
		top hit virus	accession No	family	E-value
Brown algae endornavirus 1		Bell pepper alphaendornavirus isolate Chocolate	MG545536	Endornaviridae	6.00E-09
Brown algae endornavirus 2		Riboviria sp. isolate H1_Bulk_Litter_6_scaffold_32	MN034496.1	unclassified RNA viruses	2.00E-16
Brown algae RNA virus 1		No hit			
Diatom RNA virus 1		Diatom colony associated dsRNA virus 16	NC_040310.1	unclassified RNA viruses	1.00E-47
Diatom totivirus 1	1	Zebrafish DNA sequence from clone DKEY-262J3 in linkage group 14	CT573264.9		3.00E-11
	2	Diatom colony associated virus-Like RNA	AP014917.1	unclassified RNA viruses	6.00E-13
Red algae totivirus 1	1	Diatom colony associated dsRNA virus 10	NC_040659.1	unclassified RNA viruses	2.00E-05
	2	No hit			
	3	No hit			

	BlastX					
identity	top hit virus	top hit gene	accession No	family	E-value	identity
92 %	Winged bean alphaendornavirus 1	polyprotein	YP_009305414	Endornaviridae	7.00E-115	41 %
67 %	Soybean leaf-associated endornavirus 1	RdRp	ALM62234	Endornaviridae	4.00E-113	43 %
	Hubei virga-like virus 17	RdRp	YP_009337715	unclassified RNA viruses	1.00E-70	28 %
66 %	Diatom colony associated dsRNA virus 16	RdRp	YP_009551333	unclassified viruses	0	56 %
73 %	Diatom colony associated virus-Like RNA	coat protein	BAU79523	Totiviridae	2E-133	38 %
72 %	Diatom colony associated virus-Like RNA	RdRp	BAU79524	Totiviridae	0	46 %
68 %	Thelebolus microsporus totivirus 1	RdRp	AZT88643	Totiviridae	1.00E-154	38 %
	No hit					
	No hit					
